# Vick's Floral Guide

**Published:** 1889-02

**Authors:** 


VICK'S FLORAL GUIDE.—It did seem as though the seedsmen outdid themselves last year in the line of elaborate catalogues, but here comes Vick's Floral Guide for 1889, from Rochester, N. Y., better than all previous issues. "Better" hardly expresses it—rather, we should say, far superior. It has been changed in every respect; new cuts, new type, enlarged in size (opening like an old-fashioned singing-book); contains three elegant lithographs (8x10¾ inches) of Roses, Geraniums and Melon and Tomato; besides a very fine plate of the late James Vick and his three sons who now own and manage this large business. These features must make the Floral Guide valuable to their many thousands of customers in this country.

We also notice that Vick returns to the plan started by the founder of the business years ago, of offering cash prizes at the State Fair. One would think they were a little out of their heads to offer to the public such a work as the Guide free, for that is what it amounts to, when they say it will be sent on receipt of fifteen cents, and that a certificate good for fifteen cents worth of seed will be returned with the Guide.

